# Inpatient hypoglycaemia: understanding who is at risk

**DOI:** 10.1007/s00125-020-05139-y

**Published:** 2020-04-17

**Authors:** Yue Ruan, Zuzana Moysova, Garry D. Tan, Alistair Lumb, Jim Davies, Rustam D. Rea

**Affiliations:** 1 Oxford Centre for Diabetes, Endocrinology and Metabolism, Oxford University Hospitals NHS Foundation Trust, Headington, Oxford, OX3 7LE UK; 2 Big Data Institute, University of Oxford Li Ka Shing Centre for Health Information and Discovery, University of Oxford, Oxford, UK; 3 NIHR Oxford Biomedical Research Centre, OUH, Oxford, UK

**Keywords:** Diabetes, Distribution, Hypoglycaemia, Incidence, Inpatients, Medication

## Abstract

**Aims/hypothesis:**

We analysed data obtained from the electronic patient records of inpatients with diabetes admitted to a large university hospital to understand the prevalence and distribution of inpatient hypoglycaemia.

**Methods:**

The study was conducted using electronic patient record data from Oxford University Hospitals NHS Foundation Trust. The dataset contains hospital admission data for patients coded for diabetes. We used the recently agreed definition for a level 1 hypoglycaemia episode as any blood glucose measurement <4 mmol/l and a level 2 hypoglycaemia episode as any blood glucose measurement <3 mmol/l. Any two or more consecutive low blood glucose measurements within a 2 h time window were considered as one single hypoglycaemic episode.

**Results:**

We analysed data obtained from 17,658 inpatients with diabetes (1696 with type 1 diabetes, 14,006 with type 2 diabetes, and 1956 with other forms of diabetes; 9277 men; mean ± SD age, 66 ± 18 years) who underwent 32,758 hospital admissions between July 2014 and August 2018. The incidence of level 1 hypoglycaemia was 21.5% and the incidence of level 2 hypoglycaemia was 9.6%. Recurrent level 1 and level 2 hypoglycaemia occurred, respectively, in 51% and 39% of hospital admissions in people with type 2 diabetes with at least one hypoglycaemic episode, and in 55% and 45% in those with type 1 diabetes. The incidence of level 2 hypoglycaemia in people with type 2 diabetes, when corrected for the number of people who remained in hospital, remained constant for the first 100 h at approximately 0.15 events per h per admission. With regards to the hypoglycaemia distribution during the day, after correcting for the number of blood glucose tests per h, there were two clear spikes in the rate of hypoglycaemia approximately 3 h after lunch and after dinner. The highest rate of hypoglycaemia per glucose test was seen between 01:00 hours and 05:00 hours. Medication had a significant impact on the incidence of level 2 hypoglycaemia, ranging from 1.5% in people with type 2 diabetes on metformin alone to 33% in people treated with a combination of rapid-acting insulin analogue, long-acting insulin analogue and i.v.-administered insulin.

**Conclusions/interpretation:**

Retrospective analysis of data from electronic patient records enables clinicians to gain a greater understanding of the incidence and distribution of inpatient hypoglycaemia. This information should be used to drive evidence-based improvements in the glycaemic control of inpatients through targeted medication adjustment for specific populations at high risk of hypoglycaemia.

**Electronic supplementary material:**

The online version of this article (10.1007/s00125-020-05139-y) contains peer-reviewed but unedited supplementary material, which is available to authorised users.

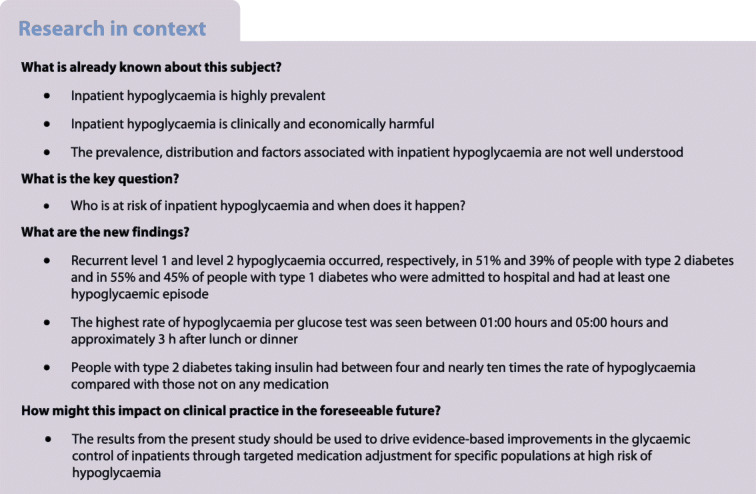



## Introduction

There is increasing recognition that hyperglycaemia and hypoglycaemia during an inpatient admission are associated with poor outcomes [1–4]. This has led to a renewed interest in achieving tight glucose control without increasing the burden of inpatient hypoglycaemia [5, 6].

However, the prevalence, distribution and factors associated with inpatient hypoglycaemia are not well understood. Despite having aggregated data for the type of diabetes and treatment with insulin or oral hypoglycaemic agent, there is a lack of detail about the timings of hypoglycaemia, the presence of recurrent hypoglycaemia and the relationship between adverse outcomes and specific medication. There is an acknowledged need to understand more comprehensively and in more detail the distribution of inpatient hypoglycaemia and the factors associated with it, in order to develop evidence-based interventions that can be implemented at scale to reduce this significant clinical burden.

This study aims to provide a detailed analysis of inpatient hypoglycaemia according to the type of diabetes during a 4 year period for over 17,000 people with diabetes in a large teaching hospital in the UK. This includes analysis of inpatient hypoglycaemia by medication type, timing of hypoglycaemia, recurrence of hypoglycaemia, age distribution and patient demographics.

## Methods

### Datasets

**Fig. 1:**
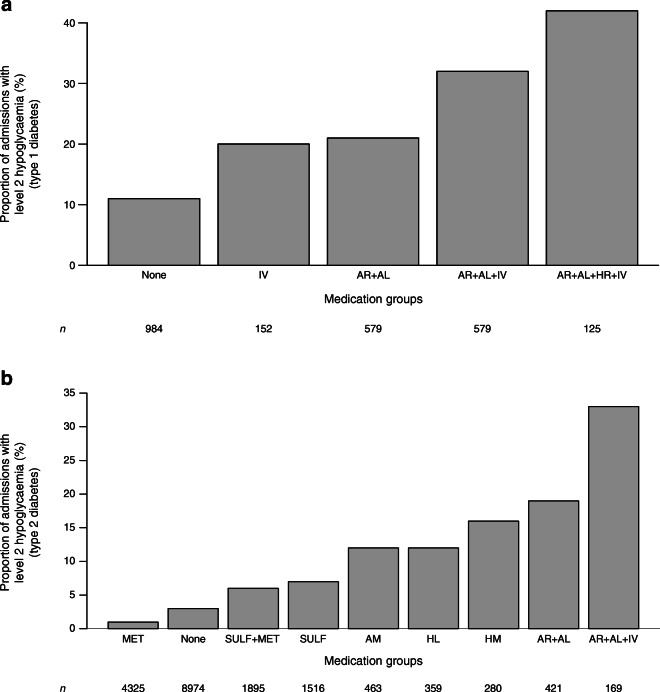
Influence of medication use on the incidence of level 2 (clinically significant) hypoglycaemia in patients with (a) type 1 diabetes and (b) type 2 diabetes. Each bar represents the proportion of admissions of patients who had level 2 hypoglycaemia and who were only prescribed the medication shown. The number of admissions for the most frequently used glucose-lowering medication groups are shown below the *x*-axis. AL, long-acting insulin analogue; AM, mixed insulin analogues; AR, rapid-acting insulin analogue; HL, long-acting human insulin; HM, mixed human insulin; HR, rapid-acting human insulin; IV, i.v.-administered insulin; MET, metformin; None, none of the medications of interest were used; SULF, sulfonylurea

The study was conducted using electronic patient record (EPR) data from Oxford University Hospital’s (OUH) NHS Foundation Trust. It was approved by the OUH Clinical Data Warehouse Programme Board following completion of a Data Protection Impact Assessment. Data was extracted from the Cerner EPR system, the laboratory information management system (LIMS) and the point-of-care testing (POCT) system. The data included all demographics, laboratory results including POCT, vital signs, medication data (only medication that was administered during the hospital admission) and procedural data. Medication taken prior to the patient’s admission was not included as this information was not available in the hospital EPR system. The blood glucose measurements used in the data analysis were the capillary blood glucose values obtained from the Abbott PXP and FPP point-of-care system as well as glucose values sent to the hospital laboratory. The dataset contained hospital admission data from 1 September 2014 to 30 June 2018 for patients with diabetes who met all of the following criteria: (1) being an inpatient as coded in the EPR; (2) having one diagnosis code among E10 (insulin-dependent diabetes mellitus), E11 (non-insulin-dependent diabetes mellitus), E13 (other specified diabetes mellitus), E14 (unspecified diabetes mellitus) or O24 (diabetes mellitus in pregnancy), as defined in the WHO International Classification of Diseases–10th Revision (ICD-10) [7]; and (3) having at least one blood glucose test performed during the hospital admissions. Data flow from the different EPR subsystems to the final dataset used for data analysis is shown in electronic supplementary material (ESM) Fig. 1.

### Hypoglycaemic episodes

A level 1 hypoglycaemic episode was defined as any blood glucose measurement <4 mmol/l and a level 2 hypoglycaemic episode was defined as any blood glucose measurement <3 mmol/l [8]. Any two or more consecutive low blood glucose measurements within a 2 h time window were considered as one single hypoglycaemic episode.

### Statistical analysis

The data was analysed according to the type of diabetes. The missingness of the variables was calculated and included in the analysis. Original distributions of the frequency of hypoglycaemic episodes vs time since hospital admission were plotted as well as the normalised distributions. These were defined as the number of hypoglycaemic episodes divided by the total number of blood glucose measurements during the defined time periods (ESM Fig. 2). Statistical analysis of baseline characteristics (mean ± SD) was performed using R version 3.3 (Vienna, Austria).

## Results

We analysed data obtained from 17,658 inpatients with diabetes (1696 with type 1 diabetes, 14,006 with type 2 diabetes and 1956 with other forms of diabetes; 9277 men; mean ± SD age, 66 ± 18 years) who underwent 32,758 hospital admissions between July 2014 and August 2018. We identified all the level 1 and level 2 hypoglycaemic episodes during these admissions. The incidence of level 1 hypoglycaemia during a hospital admission was 21.5% and of level 2 hypoglycaemia was 9.6%.

A selection of the baseline characteristics, vital signs, laboratory test results, medication use and the glycaemic outcomes for the total inpatient cohort (total cohort [TC]) and for those who had level 2 hypoglycaemia (hypoglycaemic cohort [HC]) is reported in Table 1. There was a high level of completeness of the data (sex, age and ethnicity, 100%; systolic BP and eGFR, 80%). Most variables were similar between the two groups with the main difference being the proportion of people with type 1 diabetes in the HC (21.9%) which was double that in the TC (9.6%). In relation to medication, patients in the HC were prescribed more i.v.-administered/analogue/human insulin but less metformin. There was no difference in the rate of hypoglycaemia in patients prescribed DPP-4 inhibitors and GLP-1 agonists. The mean blood glucose levels were similar between the TC and the HC.

**Table 1:** Baseline characteristics and glycaemic outcomes of the total diabetes inpatient cohort and of the inpatient cohort experiencing level 2 hypoglycaemia (blood glucose <3.0 mmol/l)

Characteristic	Inpatients with diabetes (*N* = 17,658)	Inpatients with diabetes who had level 2 hypoglycaemia (*N* = 2411)
Hospital admissions (*n*)	32,758	3154
Sex, *n* (%)		
Female	8381 (47)	1220 (51)
Male	9277 (53)	1191 (49)
Age, years	66 ± 18	64 ± 20
Ethnicity, *n* (%)		
White British	12,511 (70.9)	1751 (72.6)
African	116 (0.7)	25 (1.0)
Pakistani	331 (1.9)	33 (1.4)
Chinese	53 (0.3)	5 (0.2)
Indian	254 (1.4)	33 (1.4)
Not stated	2869 (16.2)	340 (14.1)
Other	1524 (8.6)	224 (9.3)
Type of diabetes, *n* (%)		
Type 1 diabetes	1696 (9.6)	527 (21.9)
Type 2 diabetes	14,006 (79.3)	1568 (65.0)
Other forms (including GDM)	1956 (11.1)	316 (13.1)
Systolic BP, mmHg	132.5 ± 18.2	130.3 ± 17.5
eGFR, ml min^−1^ [1.73 m]^−2^	29.8 ± 6.4	29.6 ± 6.2
Medication use, *n* (%)		
Sulfonylurea	6435 (19.6)	553 (17.5)
DPP-4 inhibitor	1415 (4.3)	128 (4.1)
GLP-1	349 (1.1)	31 (1.0)
Metformin	10,756 (32.8)	719 (22.8)
Insulin		
i.v.-administered	4678 (14.3)	1108 (35.1)
Rapid-acting analogue	3954 (12.1)	988 (31.3)
Mixed analogue	1553 (4.7)	292 (9.3)
Long-acting analogue	5118 (15.6)	1218 (38.6)
Rapid-acting human	3561 (10.9)	750 (23.8)
Mixed human	1388 (4.2)	327 (10.4)
Long-acting human	2394 (7.3)	438 (13.9)
Procedures, *n* (%)^a^	22,931 (70.0)	2431 (77.1)
Glycaemic outcomes		
Hypoglycaemia, *n* (%)		
Level 1 hypoglycaemia	7030 (21.5)	NA
Level 2 hypoglycaemia	3154 (9.6)	NA
Blood glucose, mmol/l	10.1 ± 4.7	10.2 ± 5.4

Data are presented as mean ± SD, *n* (% of total no. of patients/admissions)

^a^Number of hospital admissions with patients undergoing any type of procedure while an inpatient (based on OPCS Classification of Interventions and Procedures codes)

DPP-4, dipeptidyl peptidase-4; GDM, gestational diabetes mellitus; GLP-1, glucagon-like peptide-1

The proportion of admissions with level 2 hypoglycaemia, for the most frequently prescribed diabetes medication groups, is shown in Fig. 1 for type 1 diabetes and type 2 diabetes separately. In people with type 1 diabetes, the rate of hypoglycaemia was similar between those who were only prescribed i.v.-administered insulin and those who were only administered a combination of rapid- and long-acting insulin analogue. Those who were on a combination of insulin analogue and i.v.-administered insulin had a 50% increased risk of significant hypoglycaemia. In people with type 2 diabetes who were administered only metformin, the rate of hypoglycaemia was only 1.5% whereas for those who were being treated with a combination of rapid-acting insulin analogue, long-acting insulin analogue and i.v.-administered insulin, the rate of hypoglycaemia was as high as 33%.

Additional analysis revealed that in people with type 2 diabetes, recurrent level 1 (biochemical) and level 2 (clinically significant) hypoglycaemia occurred in 51% and 39% of hospital admissions, respectively (ESM Table 1). In people with type 1 diabetes the rates of recurrent hypoglycaemia were 55% and 45%, respectively. ESM Table 2 provides additional information for individuals with other forms of diabetes.

When the timing of hypoglycaemia was examined, the incidence of level 1 and level 2 hypoglycaemia decreased dramatically during the admission, with the majority of hypoglycaemia occurring within the first 50 h of hospital admission (ESM Fig. 2). When corrected for the number of people remaining in hospital, the rate of level 2 hypoglycaemia in people with type 2 diabetes per admission hours in hospital remained constant for the first 100 h, at 0.15 events per h per admission.

The incidence of level 1 hypoglycaemia in people with type 1 diabetes (37%) was found to be double that seen in people with type 2 diabetes (18%) and this was more marked for level 2 hypoglycaemia (ESM Fig. 3). The incidence of level 1 hypoglycaemia in people with type 1 diabetes was similar across all age groups, between 35% and 40%. This was also seen with level 2 hypoglycaemia (20–26%) (ESM Fig. 4). This age distribution pattern of hypoglycaemia was also seen in people with type 2 diabetes (ESM Fig.4).

Initial analysis of the distribution of hypoglycaemia through the day showed spikes of hypoglycaemia just before mealtimes. However, when adjusted for the number of blood tests performed each h, the distribution of level 1 and level 2 hypoglycaemia altered significantly. High levels of hypoglycaemia were seen approximately 3 h after lunch and dinner with a smaller peak 3 h after breakfast (peaks at 11:00, 16:00 and 24:00 hours; ESM Fig. 5). However, the highest rates of hypoglycaemia per glucose test were seen between 01:00 hours and 05:00 hours in people with type 1 diabetes and type 2 diabetes.

The proportion of admissions with level 1 hypoglycaemia, for the most frequently prescribed diabetes medication groups, is shown in ESM Fig. 6 for people with type 1 diabetes and type 2 diabetes.

## Discussion

This is the most detailed study of the epidemiology of inpatient hypoglycaemia published to date, analysing the prevalence of hypoglycaemia in over 17,000 patients with diabetes. The variability in the rate of hypoglycaemia was highly dependent on the medication administered but not on the duration of admission or the age of the person. Hypoglycaemia (corrected for the number of blood glucose values per h) peaked 3 h after lunch or dinner, and also between 01:00 hours and 05:00 hours. The incidences of recurrent hypoglycaemia were very high in both type 1 diabetes and type 2 diabetes.

Our data are consistent with the findings of the National Diabetes Inpatient Audit (2017), which showed a similar prevalence of level 1 hypoglycaemia and level 2 hypoglycaemia (18.4% and 7.0%, respectively) [9].

The strengths of this study include the size of the dataset and the comprehensive networked blood glucose measurements and electronic medical prescribing. This has enabled the hypoglycaemia events to be analysed according to the medication as well as the timing from admission.

The weaknesses of the study are the lack of data on the prevalence of hypoglycaemia in the community prior to admission, and the absence of other well-known risk factors for hypoglycaemia, such as hypoglycaemia awareness, carbohydrate intake, duration of diabetes and exercise, which are not captured within the EPR.

Inpatient hypoglycaemia is both clinically dangerous and economically costly: a recent study of inpatient hypoglycaemia calculated an additional length of stay of 7.1 days and a higher mortality risk (OR 1.49). The average cost of treating a patient experiencing an episode of hypoglycaemia was 40% greater than that for treating those without hypoglycaemia [10]. Therefore, a greater understanding of the factors associated with an increased risk of inpatient hypoglycaemia is vitally important in order to develop evidence-based interventions to prevent hypoglycaemia occurring while people are in hospital.

In conclusion, this retrospective analysis of data from EPRs provides a detailed clinical understanding of inpatient hypoglycaemia and lays the foundation for further work to prevent hypoglycaemia through targeting high-risk patients.

## Electronic supplementary material

ESM (PDF 2299 kb)

## Data Availability

The datasets analysed during the current study are not publicly available due to data stored in a secured data management platform but may be available from the corresponding author on reasonable request.

## Abbreviations

EPR: Electronic patient record
HC: Hypoglycaemic cohort
OUH: Oxford University Hospital
POCT: Point-of-care testing
TC: Total cohort
