# Supplementary material for: Inpatient hypoglycaemia: understanding who is at risk
Source: Diabetologia. 2020 Apr 17;63(7):1299–304. doi: 10.1007/s00125-020-05139-y (PMC7286944; doi:10.1007/s00125-020-05139-y)
Supplement: Supplementary file 1 — (PDF 2299 kb) [file 125_2020_5139_MOESM1_ESM.pdf]

Electronic supplementary material

ESM Table 1. Distribution of number of hypoglycemic episodes per admission for admissions with at least one hypoglycemic episode

Type 1 diabetes:

	1 episode	2 episodes	3 episodes	4 episodes or more
Level 1 hypoglycaemia (BG < 4mmol/L)	45%	19%	9%	27%
Level 2 hypoglycaemia (BG < 3mmol/L)	55%	18%	8%	19%

Type 2 diabetes:

	1 episode	2 episodes	3 episodes	4 episodes or more
Level 1 hypoglycaemia (BG < 4mmol/L)	49%	19%	10%	22%
Level 2 hypoglycaemia (BG < 3mmol/L)	61%	19%	8%	12%

Other forms of diabetes:

	1 episode	2 episodes	3 episodes	4 episodes or more
Level 1 hypoglycaemia (BG < 4mmol/L)	59%	18%	10%	13%
Level 2 hypoglycaemia (BG < 3mmol/L)	69%	19%	5%	7%

ESM Table 2. Comparison of baseline characteristics between the total population with diabetes and people with other forms of diabetes

Characteristics	Total inpatients with diabetes (N = 17,658) Number of hospital admissions (n = 32,758)		Inpatients with other forms of diabetes including gestational diabetes (N = 1,956) Number of admissions (n = 2,720)
Sex, N(%)			
	Female	8,381(47)	1,690(86)
	Male	9,277(53)	266(14)
Age, mean(SD)		66(18)	46(20)
Ethnicity, N(%)			
	White British	12,511(70.8)	1,315(67.2)
	African	116(0.7)	31(1.6)
	Pakistani	331(1.9)	64(3.3)
	Chinese	53(0.3)	16(0.8)
	Indian	254(1.4)	56(2.9)
	Not stated	2869(16.2)	424(21.8)
	Other	1524(8.6)	50(2.6)
Systolic blood pressure, mean(SD)		132.5(18.2)	126.3(17.5)
eGFR, mean(SD)		29.8(6.4)	29.7(6.3)
Hemoglobin, mean(SD)		29.9(6.4)	30.0(6.3)
**Medication use**			
Sulfonylurea, n(%)		6,435(19.6)	141(5.2)
DPP-4, n(%)		1,415(4.3)	23(0.8)
GLP-1, n(%)		349(1.1)	6(0.2)
Metformin, n(%)		10,756(32.8)	334(12.3)
Insulin, n(%)			
	Intravenous insulin	4,678(14.3)	231(8.5)
	Rapid analogue	3,954(12.1)	384(14.1)
	Mixed analogue	1,553(4.7)	33(1.2)
	Long analogue	5,118(15.6)	377(13.9)
	Rapid human	3,561(10.9)	112(4.1)
	Mixed human	1,388(4.2)	35(1.3)
	Long human	2,394(7.3)	125(4.6)
Procedures, n(%)		22,931(70.0)	2,243(82.4)
**Glycemic outcomes**			
Hypoglycemia, n(%)			
	Level 1 hypoglycemia	7,030(21.5)	918(33.8)
	Level 2 hypoglycemia	3,154(9.6)	350(12.9)
Blood glucose level, mean(SD)		10.1(4.7)	9.6(5.1)

N(%), number of patients and percentage over the total number of patients; n(%), number of admissions and percentage over the total number of admissions

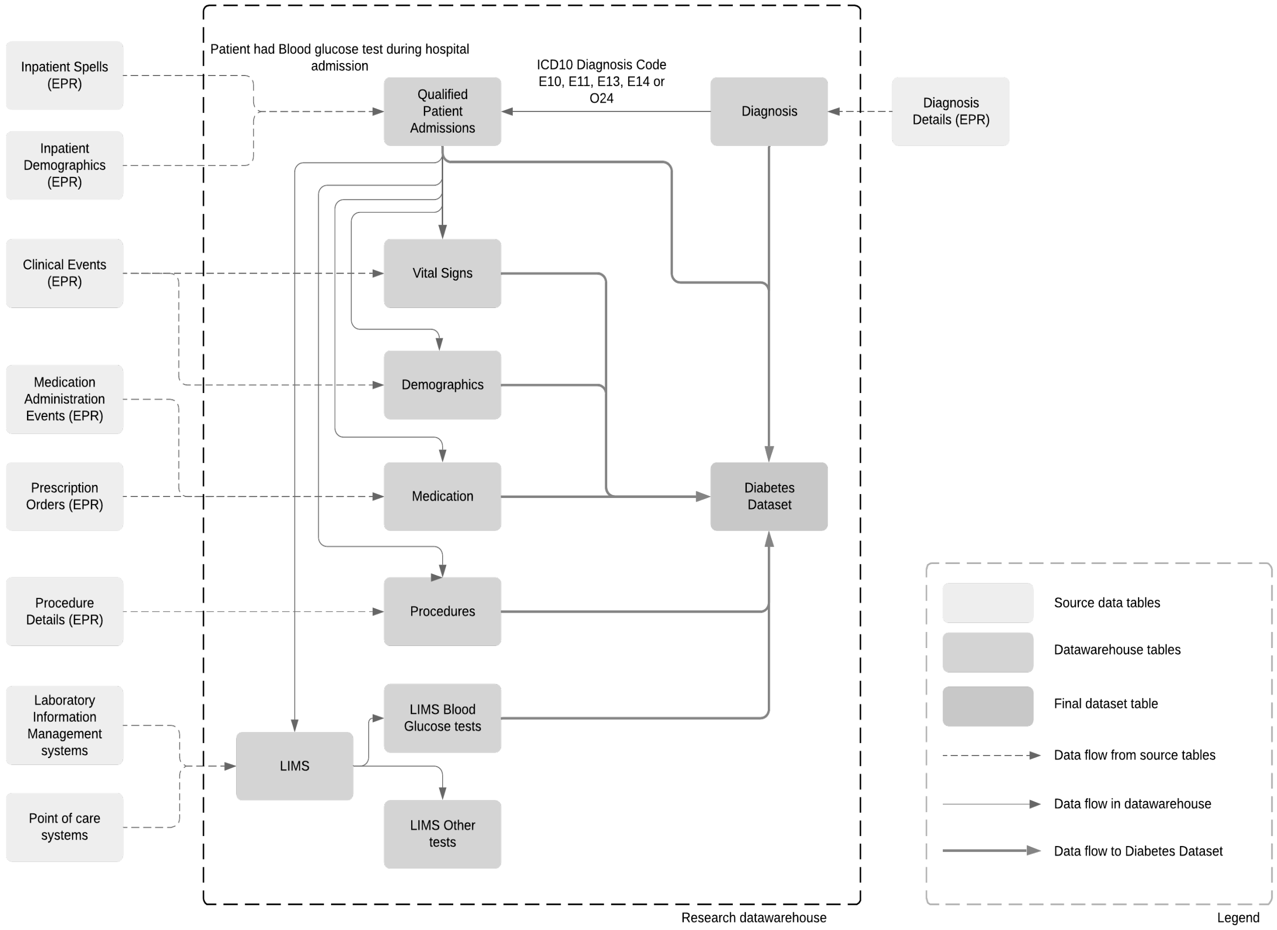

ESM Fig. 1. Data flow from the electronic health records to the final dataset used for data analysis. EPR: electronic patient records; LIMS: laboratory information management system.

Type 1 diabetes:

a.

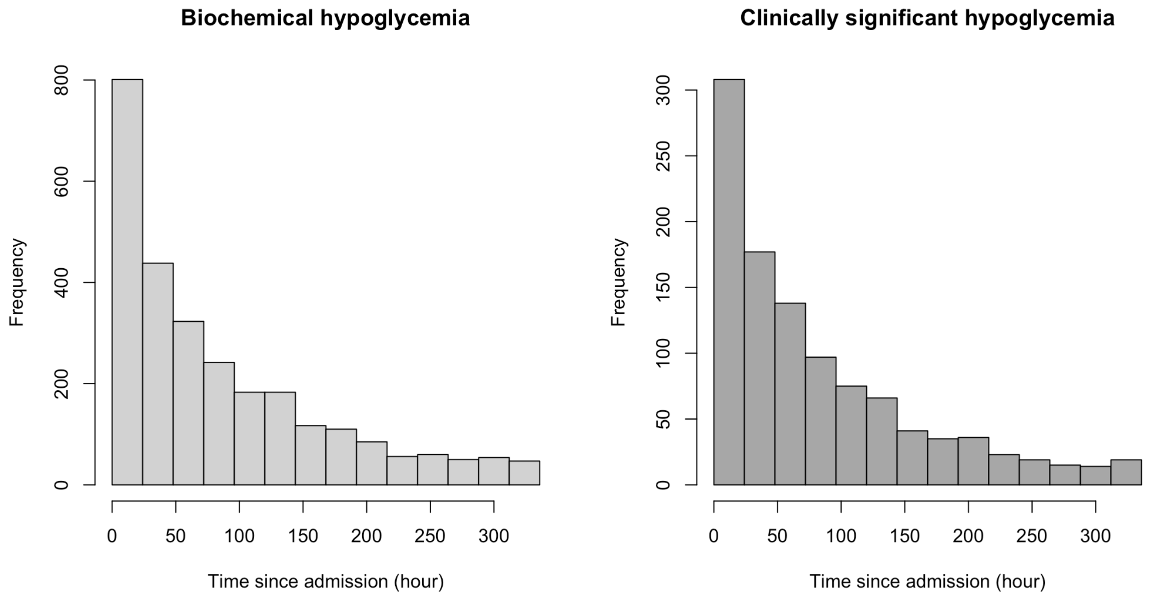

b.

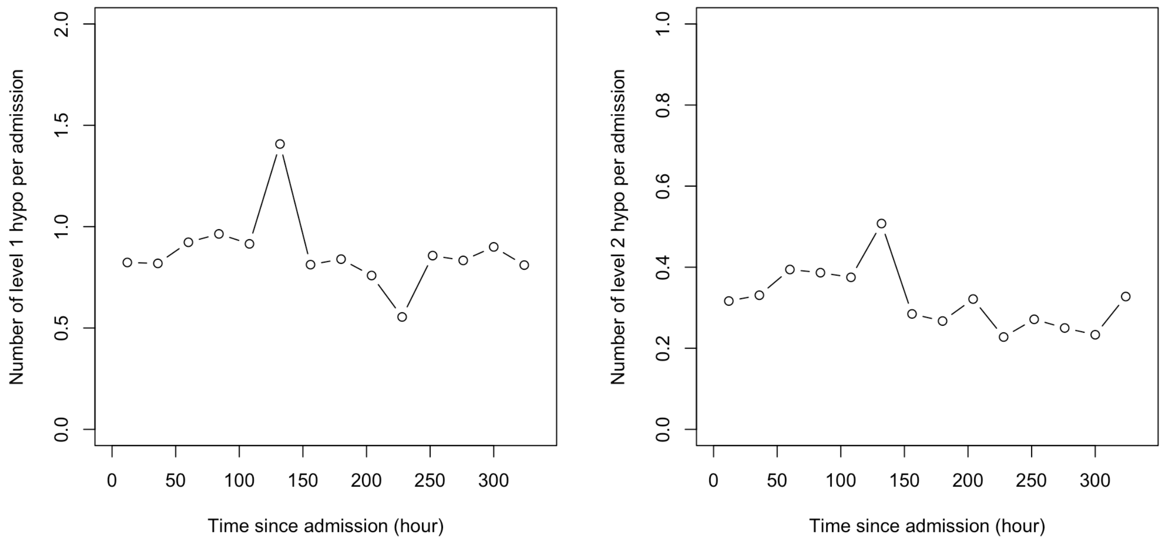

Type 2 diabetes:

c.

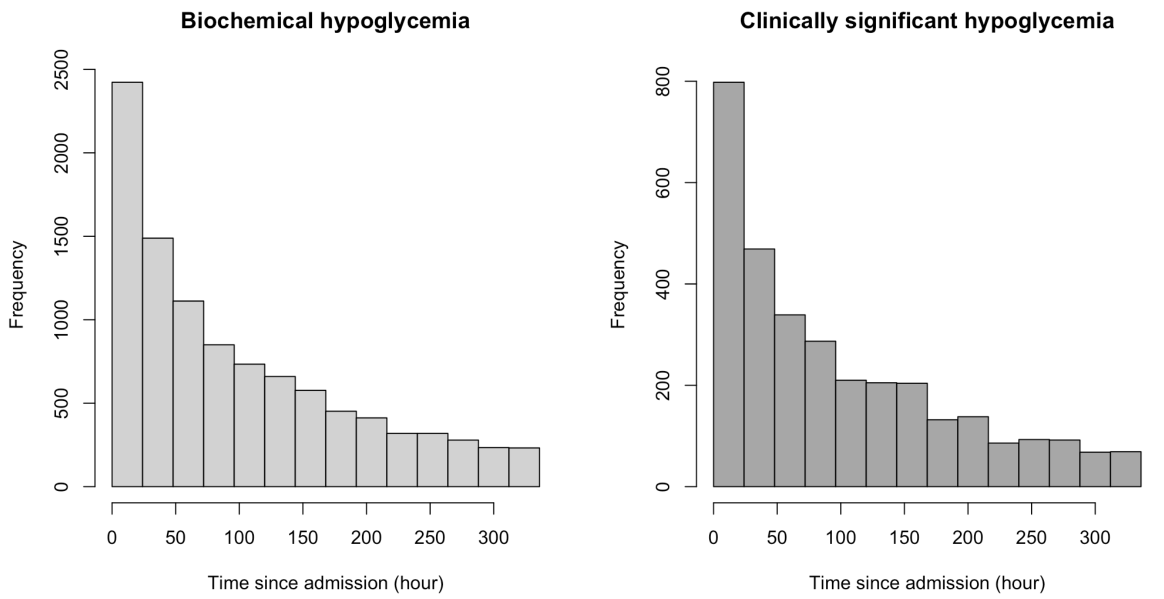

d.

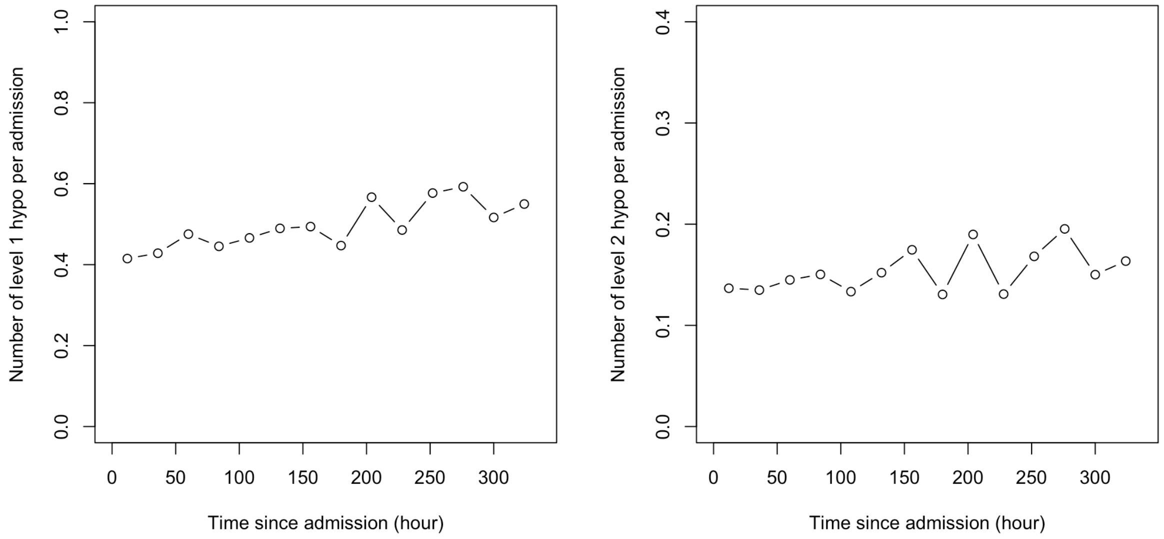

Other forms of diabetes:

e.

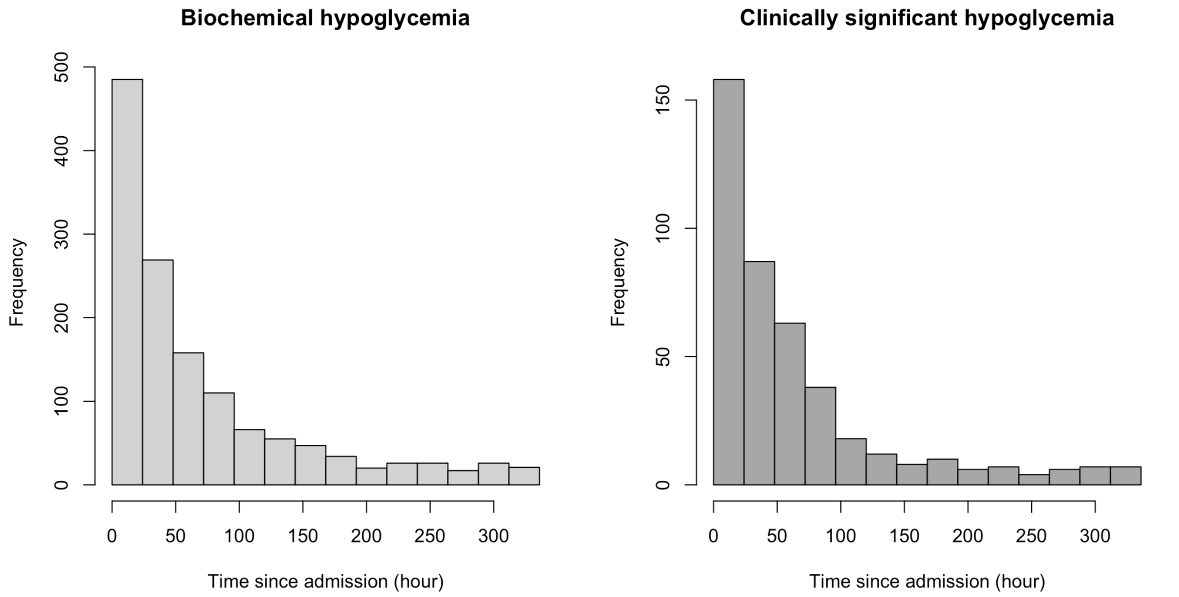

f.

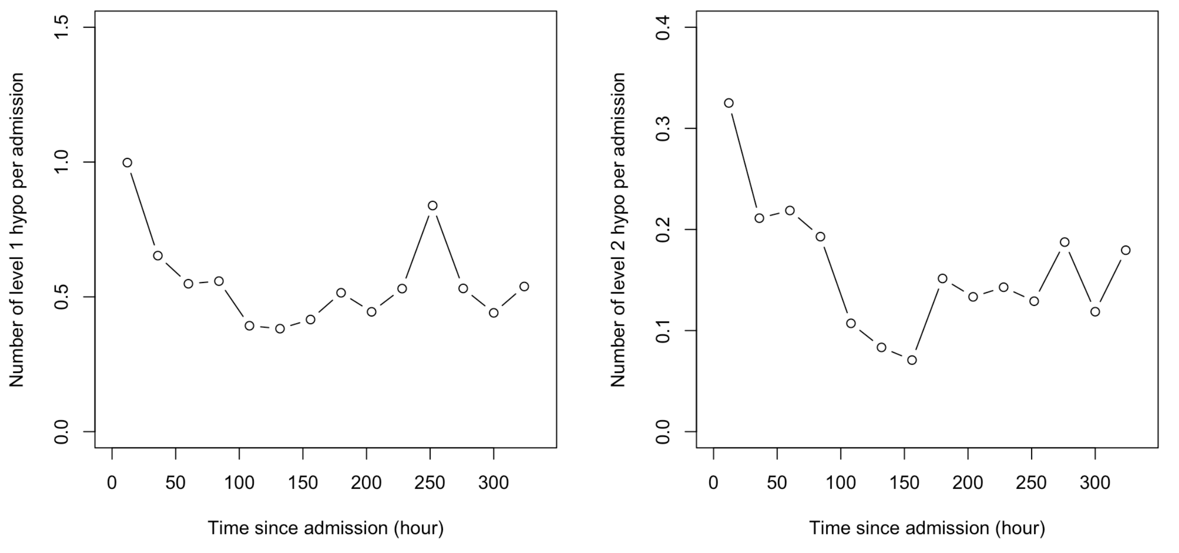

ESM Fig. 2. Distribution of the number of level 1/level 2hypoglycaemic episodes per hour within the first 14 days of admission (a, c, e). Number of hypoglycaemic episodes adjusted for number of admissions is shown in (b, d, f).

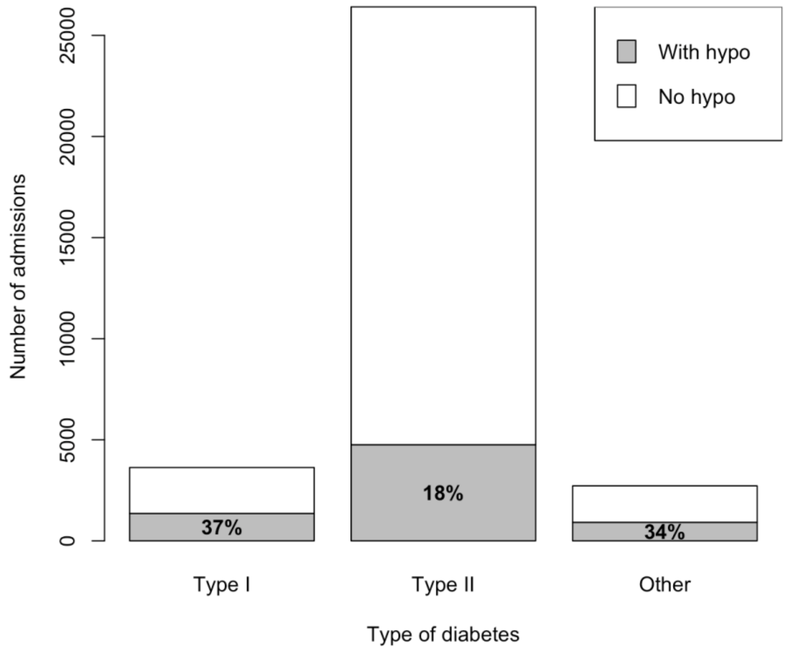

a.

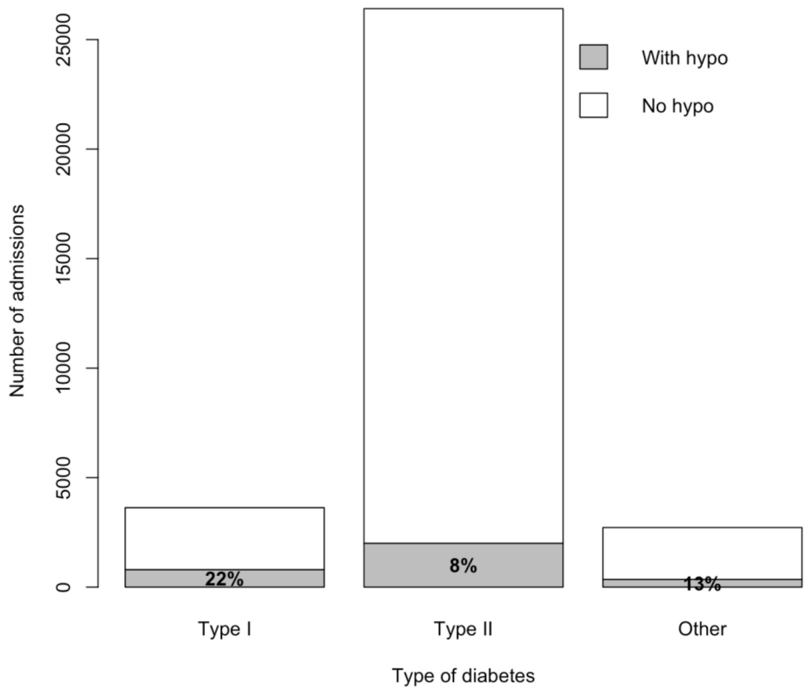

b.

ESM Fig. 3. Type of diabetes distribution of inpatient admissions with/without level 1 (a) and level 2 (b) hypoglycaemia (proportions of admissions with hypoglycaemia in each diabetes type are shown in the grey area).

Type 1 diabetes:

a.

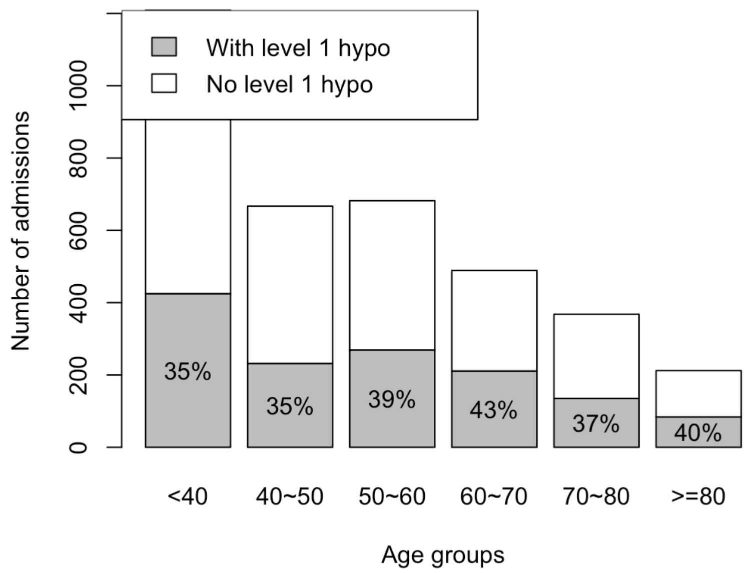

b.

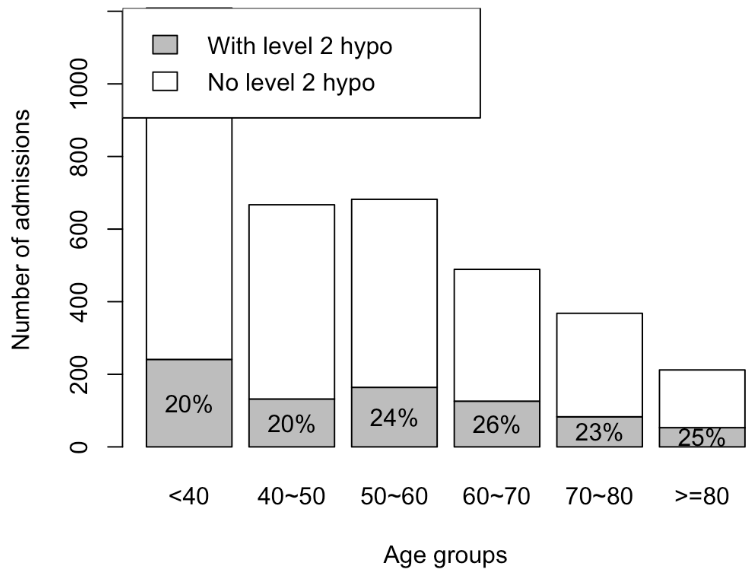

Type 2 diabetes:

c.

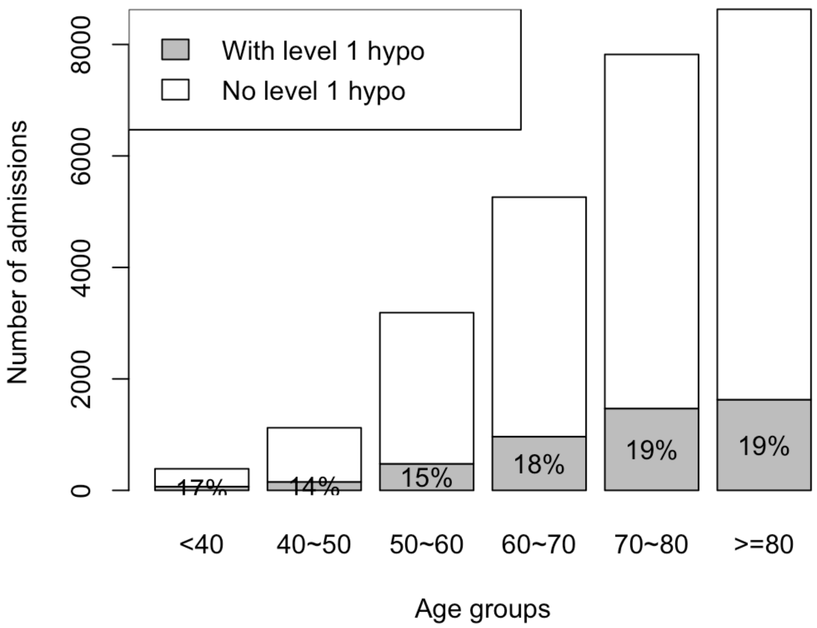

d.

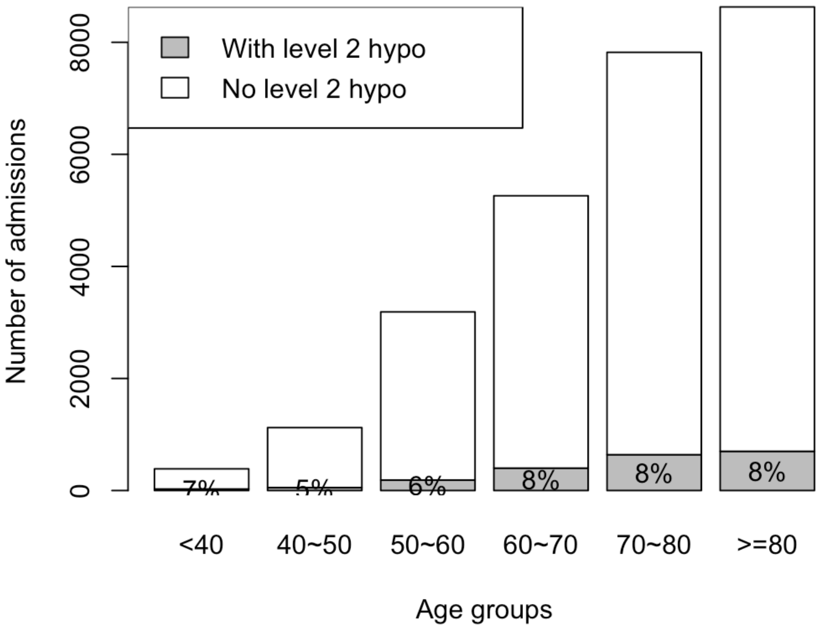

Other forms of diabetes:

e.

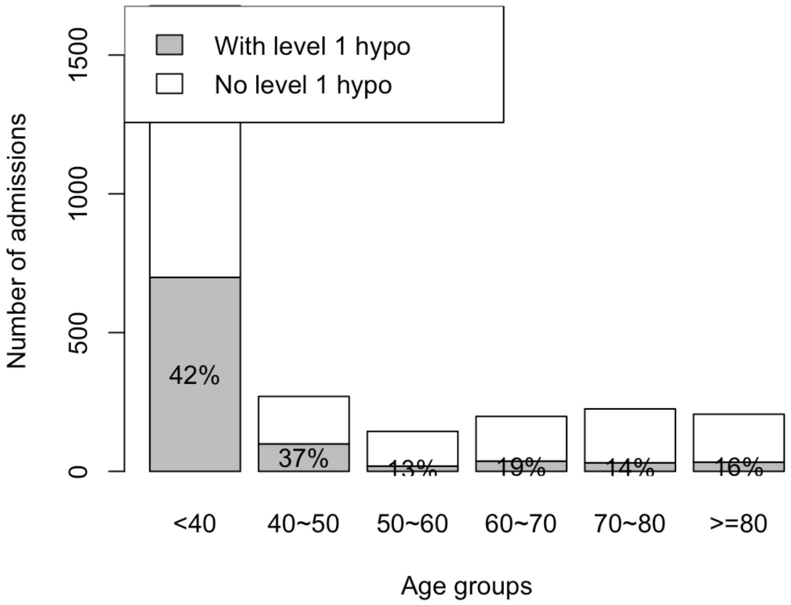

f.

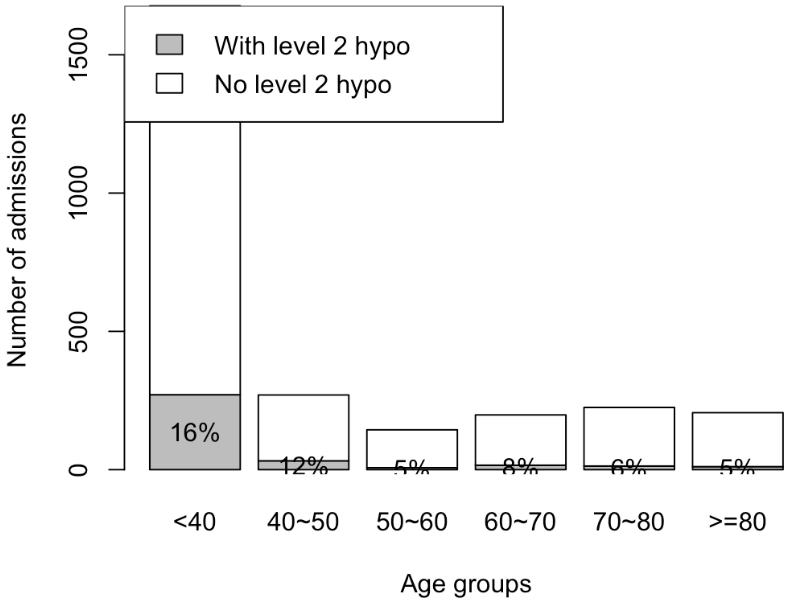

ESM Fig. 4. Age group distribution of inpatient admissions with/without level 1(a, c, e)/level 2(b, d, f) hypoglycaemia (proportions of admissions with hypoglycaemia in each age groups are shown in the grey area).

Type 1 diabetes:

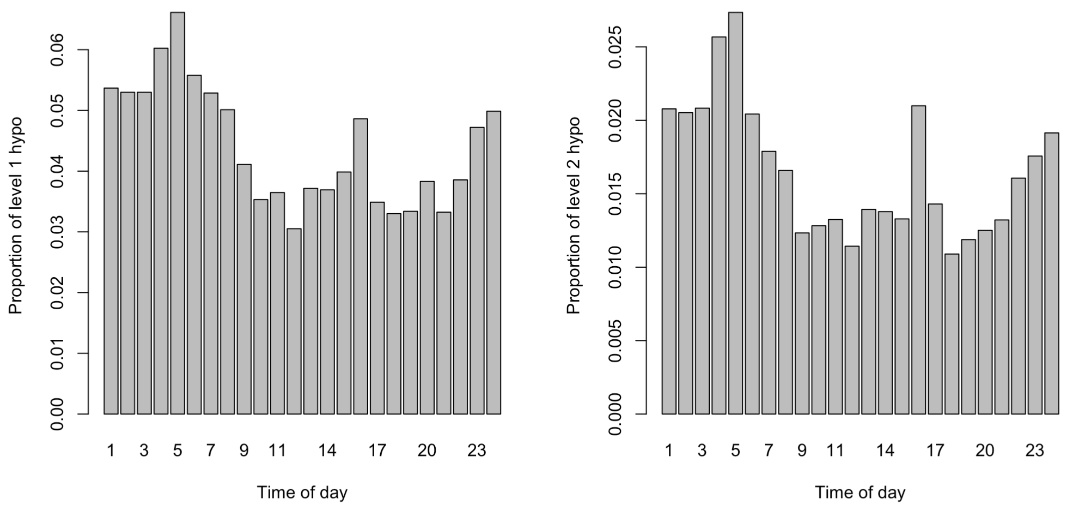

Type 2 diabetes:

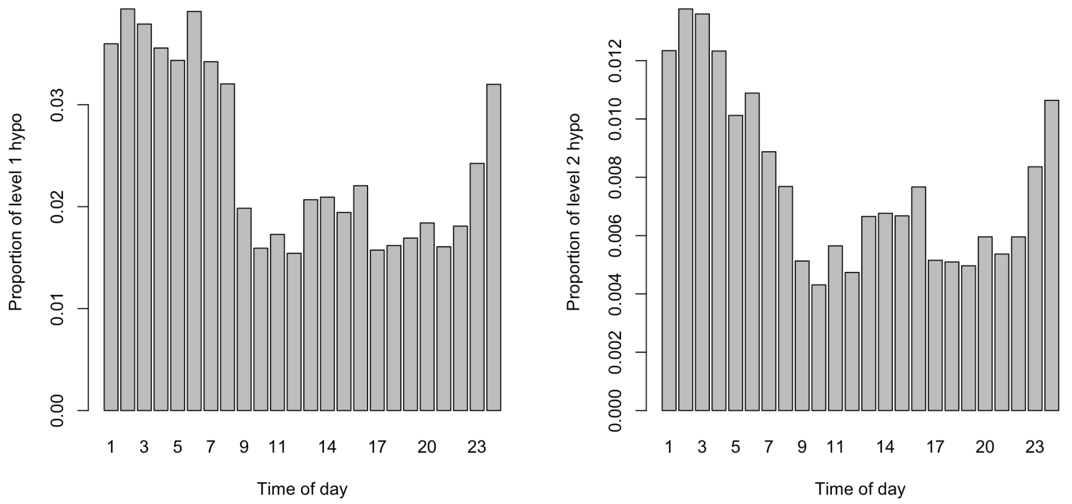

Other forms of diabetes:

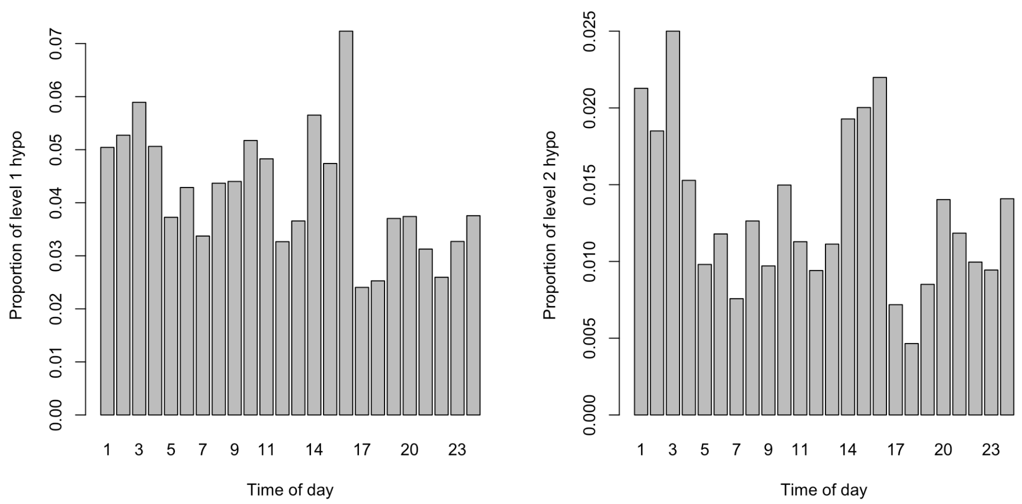

ESM Fig. 5. Time of day distribution of the number of level 1/level 2 hypoglycaemic

Type 1 diabetes:

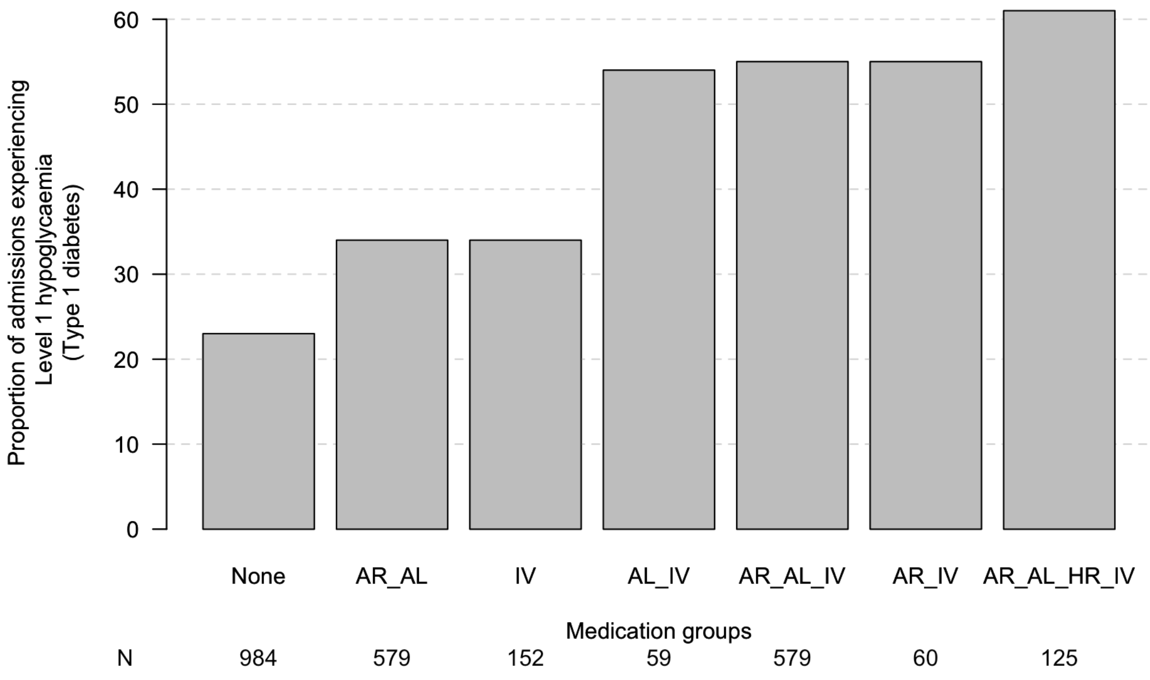

Type 2 diabetes:

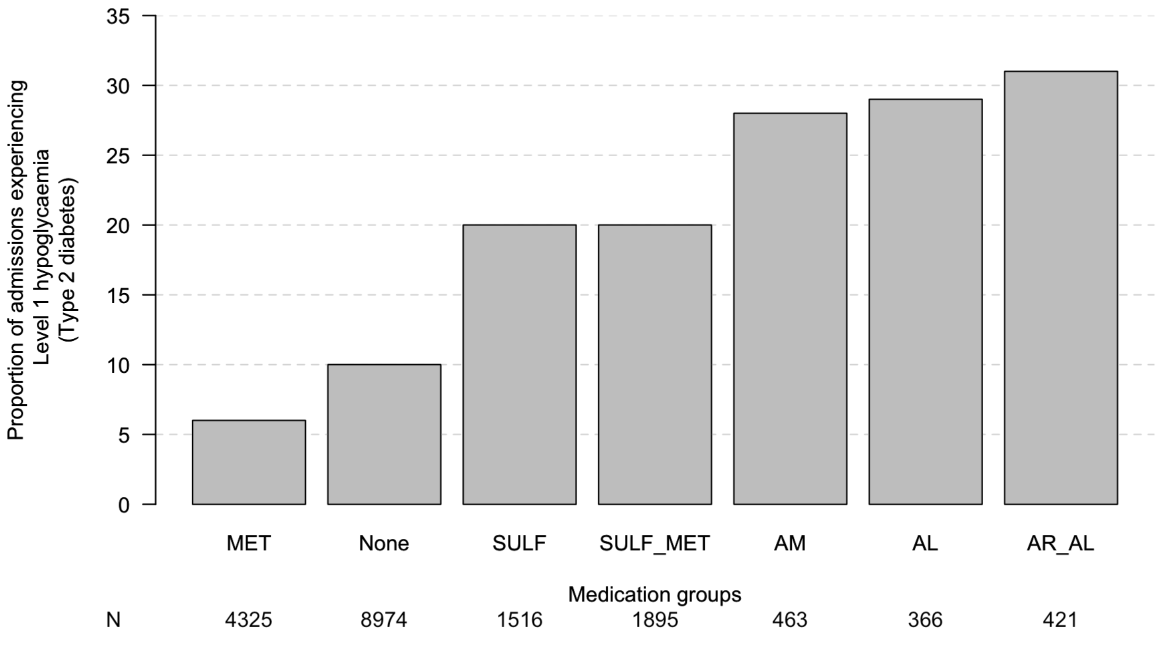

ESM Fig. 6. Influence of medication use on incidence of level 1 (biochemical) hypoglycaemia (number of admissions for the most frequently used diabetes medication groups are shown below the x-axis [N]). MET: metformin; SULF: sulfonylurea; AM: insulin analogue mixed; HL: insulin human long; AL: insulin analogue long; IV: intravenous insulin; AR: insulin analogue rapid; HR: insulin human rapid. None: none of the medications of interest was used. Each bar represents the proportion of admissions of patients who had level 1 hypoglycaemia and who were only prescribed that specific medication.
